# Predicting greater sage‐grouse habitat selection at the southern periphery of their range

**DOI:** 10.1002/ece3.6950

**Published:** 2020-10-28

**Authors:** Simona Picardi, Terry Messmer, Ben Crabb, Michel Kohl, David Dahlgren, Nicki Frey, Randy Larsen, Rick Baxter

**Affiliations:** ^1^ Jack H. Berryman Institute, Department of Wildland Resources Utah State University Logan UT USA; ^2^ Warnell School of Forestry and Natural Resources University of Georgia Athens GA USA; ^3^ Department of Plant and Wildlife Sciences Brigham Young University Provo UT USA

**Keywords:** *Artemisia* spp., *Centrocercus urophasianus*, greater sage‐grouse, habitat selection, leks, radio‐telemetry, random forest, sagebrush ecosystems, species distribution, Utah

## Abstract

Mapping suitable habitat is an important process in wildlife conservation planning. Species distribution reflects habitat selection processes occurring across multiple spatio‐temporal scales. Because habitat selection may be driven by different factors at different scales, conservation planners require information at the scale of the intervention to plan effective management actions. Previous research has described habitat selection processes shaping the distribution of greater sage‐grouse (*Centrocercus urophasianus*; sage‐grouse) at the range‐wide scale. Finer‐scale information for applications within jurisdictional units inside the species range is lacking, yet necessary, because state wildlife agencies are the management authority for sage‐grouse in the United States. We quantified seasonal second‐order habitat selection for sage‐grouse across the state of Utah to produce spatio‐temporal predictions of their distribution at the southern periphery of the species range. We used location data obtained from sage‐grouse marked with very‐high‐frequency radio‐transmitters and lek location data collected between 1998 and 2013 to quantify species habitat selection in relation to a suite of topographic, edaphic, climatic, and anthropogenic variables using random forest algorithms. Sage‐grouse selected for greater sagebrush (*Artemisia* spp.) cover, higher elevations, and gentler slopes and avoided lower precipitations and higher temperatures. The strength of responses to habitat variables varied across seasons. Anthropogenic variables previously reported as affecting their range‐wide distribution (i.e., roads, powerlines, communication towers, and agricultural development) were not ranked as top predictors at our focal scale. Other than strong selection for sagebrush cover, the responses we observed differed from what has been reported at the range‐wide scale. These differences likely reflect the unique climatic, geographic, and topographic context found in the southern peripheral area of the species distribution compared to range‐wide environmental gradients. Our results highlight the importance of considering appropriateness of scale when planning conservation actions for wide‐ranging species.

## Graphical abstract

We used random forest algorithms to map seasonal habitat selection for greater sage‐grouse at the southern periphery of their range. Results will provide scale‐appropriate information for management decisions at the state jurisdiction level in Utah.

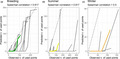

## 1 INTRODUCTION

Accurate information on the spatial distribution of imperiled wildlife species at different geographical scales is essential for effective conservation planning (Lawler et al., 2011; Rodríguez et al., 2007). Species distributions reflect habitat selection processes at multiple spatio‐temporal scales (D. H. Johnson, 1980). At the broadest scale, the distribution of species is driven by variation in the ecological and environmental conditions that may constrain species occurrence, such as climatic conditions that exceed physiological tolerance limits (Kearney & Porter, 2009). Within a species’ range, populations select for habitats that satisfy their requirements, such as preferred vegetation types (Hirzel & Lay, 2008). In turn, within these habitats, individual habitat selection patterns may be driven by environmental factors that vary at finer scales, such as seasonal fluctuations in forage abundance (Pape & Löffler, 2015). Features that are important in driving species distribution may not transfer across scales, especially when the spectrum of available conditions varies between scales (Schneider, 2001).

Effective conservation planning requires information on species distribution that matches the scale of intervention (Ferraz et al., 2012). For example, in the United States (U.S.), federal regulatory agencies such as the U.S. Fish and Wildlife Service (USFWS) routinely use species distribution models to inform decisions regarding the listing of species for protection under the U.S. Endangered Species Act (ESA; Noss et al., 1997). Species distribution models provide broadscale characterization of suitable habitat for a target species and can be used to predict and map species occurrence over large geographical areas and project persistence under future scenarios (Lawler et al., 2011; Schwartz, 2012).

In the United States, most wildlife species conservation and management decisions are made by state wildlife agencies (Organ et al., 2012). These decisions often require the identification of priority habitats within specific areas of the species range, which is achieved through finer‐scale estimates of habitat selection (e.g., Gehring & Potter, 2005; Hatten et al., 2005; Stralberg et al., 2011).. Habitat associations that are important at broadscales might not hold true at the scales important for state managers, and vice‐versa, the drivers that are important at these finer scales may not emerge from range‐wide analyses (Schneider, 2001). This is especially true for areas located at the periphery of a species’ range, where the spectrum of available environmental conditions encompasses only a subset of the full array observed across the range and thus observed habitat selection responses may be idiosyncratic (Dow, 1969; Hellmann et al., 2008; Murphy and Lovett‐Doust, 2007).

In this study, we present an analysis of habitat selection for greater sage‐grouse (*Centrocercus urophasianus*; sage‐grouse) at the southern periphery of their range, to provide information for managers at the state level in Utah. Historical information on sage‐grouse distributions has provided a comprehensive understanding of the factors that drive habitat selection over broad geographical scales (i.e., first‐order habitat selection; sensu Johnson, 1980). Aldridge et al. (2008) integrated data from multiple sources to identify land features that distinguish sites where sage‐grouse has been extirpated within their historical range from sites where they persisted. They identified key features that, when lost or fragmented, resulted in sage‐grouse local extirpation. Knick et al. (2013) incorporated range‐wide information on seasonal breeding ground (i.e., lek) locations and persistence with environmental data from remote sensing to quantify thresholds of ecological factors limiting the occurrence of the species. Sage‐grouse are an obligate sagebrush (*Artemisia* spp.) species, and thus, it was not surprising that Knick et al. (2013) found the most important factor that explained sage‐grouse distribution was the presence of contiguous sagebrush cover across the landscape. Additionally, low levels of anthropogenic disturbance, development, forest, grassland, and agriculture were important predictors of sage‐grouse occurrence at broad scales (Aldridge et al., 2008; Knick et al., 2013).

Factors that affect sage‐grouse distribution at the range‐wide scale are likely to differ, at least partially, from features that influence habitat selection at finer spatial scales (Dahlgren et al., 2019). The need for finer‐scale information on sage‐grouse habitat selection became more apparent in 2015, when the USFWS decided against listing sage‐grouse for ESA protection and confirmed state wildlife management agencies as the ongoing authority for sage‐grouse conservation (USFWS, 2015). In the decision, the USFWS reiterated that sustaining sage‐grouse populations would require better delineation of seasonal habitats in different regions within the sage‐grouse range, that is, at the scale at which state authorities operate (USFWS, 2015). In particular, an improved understanding of habitat selection in peripheral areas of the range will be important to ensure connectivity between peripheral and core populations (Cross et al., 2018; USFWS, 2015).

Information on factors affecting sage‐grouse distribution at a relevant scale for management intervention from state wildlife agencies is often limited. Range‐wide estimates are not appropriate to predict responses at finer scales, and studies focused on the scale of single populations may not prove general enough to support decision‐making at the state level. Information at an intermediate scale (corresponding to second‐order habitat selection, sensu Johnson, 1980), that refine range‐wide habitat selection estimates within the specific environmental context of a region without being specific to a single population, would be appropriate to direct management actions from state jurisdictions. Previous existing studies that have mapped sage‐grouse distributions in specific regions within the species range did not link distribution to environmental factors (e.g., Beck et al., 2003; Leonard et al., 2000; Schroeder et al., 2000).

Utah is located at the southern periphery of the sage‐grouse range (Schroeder et al., 2004). The spectrum of ecological and environmental conditions available to sage‐grouse in Utah encompasses the tails of the general distribution observed across the range (Stiver, 2006). For example, occupied sagebrush habitats in Utah consist of discontinuous patches at higher elevations than those found in other areas of the sage‐grouse range (Dahlgren et al., 2016, 2019). Climatic regimes also differ in Utah as compared to other areas of the sage‐grouse range, with generally lower annual precipitation (Dahlgren et al., 2016; West, 1983). In response to these differences in the spectrum of available conditions, sage‐grouse may select habitat differently than they do on average across their range.

We used data on lek locations and from very‐high‐frequency (VHF) telemetry between 1998 and 2013 to model seasonal habitat selection for sage‐grouse in Utah using random forest algorithms (Breiman, 2001; Cutler, 2005). We examined sage‐grouse habitat selection in relation to a suite of topographic, edaphic, climatic, and anthropogenic factors that we hypothesized would be important based on previous research (Connelly et al., 2000; Knick et al., 2013). The resulting predictive maps will support management decisions for habitat prioritization at the state level.

## 2 METHODS

### 2.1 Study area

The topography of Utah is variable, with most of the state considered mountainous. Temperatures vary with altitude and latitude. Precipitation also varies greatly, from an average of less than 12 cm annually over the Great Salt Lake Desert to more than 100 cm in the mountains (https://wrcc.dri.edu/narratives/UTAH.htm). Our study area encompassed known sage‐grouse breeding habitats and distributions in Utah (Dahlgren et al., 2016, 2019). For conservation and management purposes, the state of Utah has designated 11 sage‐grouse management areas (SGMAs; Utah Public Lands Policy Coordination Office [PLPCO], 2019). Sage‐grouse in Utah occupy a diversity of sagebrush communities from shrub‐dominated semi‐deserts in the southwest to more perennial grass‐dominated sagebrush steppe in the northeast part of the state. Big sagebrush (*A. tridentata*) varieties typically dominate most sagebrush landscapes with Wyoming (*A. t. wyomingensis*), basin (*A. t. tridentata*), and mountain (*A. t. vaseyana*) big sagebrush at lower, mid, and high elevations, respectively. Shallow soils support low (*A. arbuscula*) and black (*A. nova*) sagebrush communities throughout the state.

### 2.2 Data collection

We used data on active lek locations surveyed between 1998 and 2013 (Utah Division of Wildlife Resources [UDWR], unpublished data). The UDWR defined active leks on an annual basis from 1998 to 2013 as those leks where at least one strutting male was observed (PLPCO, 2019). From the 484 historic lek locations in the UDWR database, we identified leks that were active during the study period.

We obtained seasonal location data from VHF radio‐marked sage‐grouse in Utah between 1998 and 2013 from a database maintained by Utah State University's (USU) Community‐Based Conservation Program (USU, unpublished data). The telemetry database included locations collected by researchers at USU and Brigham Young University (BYU). During these studies, sage‐grouse were captured, marked with VHF radio‐collars, released on‐site, and routinely monitored to assess vital rates and habitat use using standard protocols (Connelly et al., 2000). Field protocols for each study were reviewed and approved by the USU or BYU Institutional Animal Use and Care Committee (see Dahlgren et al., 2016 for protocol numbers). In all cases, the UDWR approved Certificates of Registration permitting sage‐grouse captures, radio‐marking, and monitoring. The dataset included individuals from every known sage‐grouse population across the state of Utah.

### 2.3 Sampling design

We followed a use‐availability design (Manly et al., 2002) to model seasonal sage‐grouse habitat selection. We modeled each season separately because we expected sage‐grouse to select habitat differently during breeding, summer, and winter at our spatial scale of interest (Dahlgren et al., 2016, 2019). Therefore, we assigned sage‐grouse used locations to breeding, summer, and winter seasons in accordance with Utah‐specific breeding‐date ranges identified by Dahlgren et al. (2016). The breeding season included VHF locations from 1st April to 31st May as well as active lek locations; VHF locations between 1st June and 31st August were assigned to summer and those between 15th November and 15th March to winter. Because VHF locations are collected with a time lag of several days, spatio‐temporal autocorrelation due to the underlying movement process was not a concern in our dataset and we were able to retain every used point for further analysis.

We randomly generated available points across Utah to compare to known sage‐grouse use locations (Manly et al., 2002). Because habitat selection may be affected by annual variation of environmental conditions as well as variation between sites across the study area, we generated available points stratifying by year and nearest population (Barbet‐Massin et al., 2012; Buskirk & Millspaugh, 2006). We sampled available points in proportion to the number of used points in each stratum because it allowed us to control for the effect of variation across years and sites by including them as covariates in the model. Adding covariates that account for spatial or temporal structure in the data is a common approach to control for spatio‐temporal autocorrelation (Boyce, 2006). We attributed sites to used points using Voronoi polygons (D. G. Evans & Jones, 1987) around the centroids of different populations (i.e., populations inhabiting study areas showing marked differences in terms of environmental characteristics, and/or separated by land where sage‐grouse do not occur) and then generated an equal number of available points within each polygon (Appendix). Matching the number of used points in each site with an equal amount of available points also helped us neutralize the potential effect of sampling imbalance between sites.

### 2.4 Environmental variables

We intersected used and available locations with land cover, topographic, edaphic, climatic, and anthropogenic variables relevant to sage‐grouse ecology (Connelly et al., 2000). We used a 1‐km grain size (sensu Meyer & Thuiller, 2006) to intersect used locations with each environmental variable. This grain size is appropriate to analyze second‐order habitat selection for sage‐grouse, whose home‐range size ranges between 1 and 29 km^2^ in the breeding season, 26 km^2^ in the summer, and 195 km^2^ in the winter (Connelly et al., 2011).

We measured land cover variables using existing vegetation type data layers from the Landscape Fire and Resource Management Planning Tools project (Landfire; www.landfire.gov). We associated locations with values from the most recent Landfire data layer relative to the year the location was recorded. We used Landfire versions 1.0.5, 1.1.0, 1.2.0, and 1.3.0 to describe land cover for years 2001, 2008, 2010, and 2012, respectively (Table 1). Similar to Knick et al. (2013), we calculated the proportions of land cover by classification within a 5‐km‐radius circular buffer using 30‐m resolution Landfire data and then resampled to 1‐km resolution for use in habitat models (we used the *resample()* function in the R package *raster* for nearest‐neighbor resampling; Hijmans 2020). The 5‐km buffer used to summarize these covariates at each 1‐km pixel is meant to account for the fact that sage‐grouse select habitat based on the broader landscape context surrounding each location. Land cover types extracted for use in modeling included sagebrush, agriculture, grassland, riparian, conifer, and development (Table 1). Topographic variables were also obtained from Landfire (version 1.3.0) and included elevation and slope. From the digital elevation model, we also calculated a vector ruggedness measure layer following a procedure outlined by Sappington et al. (2007).

**TABLE 1:** Land cover category definitions using LANDFIRE existing vegetation types data (Landfire; www.landfire.gov)

Landcover type	LANDFIRE criteria
Big sagebrush shrubland^a^	Inter‐Mountain Basins Big Sagebrush Shrubland
Big sagebrush steppe^a^	Inter‐Mountain Basins Big Sagebrush Steppe
	Inter‐Mountain Basins Montane Sagebrush Steppe
Low sagebrush^a^	Colorado Plateau Mixed Low Sagebrush Shrubland
	Wyoming Basins Dwarf Sagebrush Shrubland and Steppe
	Great Basin Xeric Mixed Sagebrush Shrubland
	Columbia Plateau Low Sagebrush Steppe
Mountain sagebrush^a^	Artemisia tridentata ssp. vaseyana Shrubland Alliance
Agricultural	*EVT Group Physiognomy* = “Agricultural”
Conifer	*EVT Group Physiognomy* = “Conifer”
Developed	“Developed – Low,” “ – Medium,” and “ – High” Intensity classes
Grassland	*EVT Group Physiognomy* = “Grassland”
Riparian	*EVT Group Physiognomy* = “Riparian”

^a^ Included in overall sagebrush category.

Edaphic parameters reflect suitability for vegetation cover types and may be a useful indicator of resilience of sagebrush vegetation cover (Chambers et al., 2014; Maestas et al., 2016). We obtained edaphic variables describing soil to include percent clay, silt, sand, available water capacity, salinity, and soil depth from the Digital General Soil Map of the United States (STATSGO2; Natural Resources Conservation Service 2014).

The availability of forage for sage‐grouse is affected by precipitation and temperature (Connelly et al., 2011; Connelly et al., 2000; Guttery et al., 2013). Thus, we expected climatic variables to be important drivers of habitat selection in all seasons. We obtained 30‐year average precipitation, minimum, and maximum temperatures in each season by combining monthly data at 800‐m resolution from PRISM (Daly, 2002) and resampling to 1‐km resolution. To match our definition of seasons, we used monthly climate data for April and May for the breeding season, June through August for the summer, and November through March for the winter.

Nelle et al. (2000), Byrne (2002), and Crawford et al. (2004) reported that sage‐grouse may avoid burned areas for up to 20 years. We measured areas burned by wildfires as the proportion of area within a 5‐km‐radius circular buffer that burned within the last 20 years using polygons from the U.S. Geological Survey (2011, 2014). Because wildfires usually occur in late summer, we assigned values of burned area with a one‐year lag.

We obtained secondary road, state and federal highway, and interstate highway data layers from SAGEMAP (www.sagemap.wr.usgs.gov, Knick & Schueck, 2002). We combined interstate and highway road classes into a single class. Power lines and tall structures such as communication towers represent potential threats to sage‐grouse, as they provide perches for corvids and raptors (Prather & Messmer, 2010). Power line location data were obtained from SAGEMAP and from PacifiCorp, Garkane, and Raft River electric utility companies, which granted permission to use these data for the specific purposes of this research (Kohl et al., 2019). We used the Line Density tool in ArcGIS to convert linear features to a 1‐km resolution raster of line density values. The Line Density tool calculates a magnitude‐per‐unit area from line features that fall within a radius (in our case, 5 km) around each cell.

### 2.5 Data analysis

We used random forest algorithms to model sage‐grouse habitat selection in each season. While logistic regression is usually the tool of choice in habitat selection studies (Boyce et al., 2002; Keating & Cherry, 2004), random forest has been successfully used as an analytical approach to model species distributions (Evans et al., 2011; Li & Wang, 2013). Nonparametric algorithms such as random forest require no assumptions on data distribution, they are robust to correlation among explanatory variables, and they are good at capturing complex nonlinear interactions, such as threshold effects, that are difficult to model using parametric approaches (Carvalho et al., 2018; Shoemaker et al., 2018). These characteristics explain the success of random forest as a species distribution modeling approach: at the broad scales that these studies focus on, responses to predictors are often nonlinear as a result of the breadth of the environmental gradient (Huisman et al., 1993; Oksanen & Minchin, 2002). Moreover, while parametric methods like generalized linear regression are more suited for the purpose of ecological inference, machine learning approaches usually perform better in the realm of prediction (Evans et al., 2011). Therefore, despite our scale of interest being finer than a canonical species distribution model, we chose random forest as our analytical tool because our objective was to achieve high power in predicting sage‐grouse habitat across the landscape.

We fitted random forest models to data for each season separately using the R package *randomForest* (Liaw & Wiener, 2015). The response variable was, use versus availability, modeled as a function of a set of 26 predictors including the environmental variables listed above plus year and site. Our sampling protocol ensured balance between classes of the response (i.e., 1:1 ratio between used and available points), to avoid biases in classification accuracy (J. S. Evans et al., 2011). We verified that the resulting amount of available points adequately captured the spectrum of variation in environmental conditions observed across our study area (Appendix). We grew 1,000 trees for each model (*ntree* = 1,000) and set the minimum node size to 1 (*nodesize* = 1; this is the default value for classification). We performed a sensitivity analysis on the hyperparameter *mtry*, that is the number of variables used for splitting at each node; we chose the value that minimized out‐of‐bag errors and set *mtry* = 5 (default was *mtry* = sqrt(number of predictors) =~ 5). We used spatial K‐fold cross‐validation to evaluate model performance by fitting a set of models to subsets of each full seasonal dataset obtained withholding data for one site at a time. Unlike nonspatial validation methods such as random splitting of training and testing data (with or without cross‐validation), this approach accounts for spatial autocorrelation in the data and returns a more appropriate estimation of predictive power (Ploton et al., 2020). We estimated both out‐of‐bag and validation data error rates by summing confusion matrices across folds. We only calculated classification error rates for the used class because under a presence‐background study design available points are not necessarily unused, rather, their status is unknown; therefore, estimating a classification error for those does not make sense (Fieberg et al., 2018). For the same reason, standard evaluation metrics for random forest models, such as the AUC (area under the receiver operator characteristic curve; Brown & Davis, 2006), are also not appropriate in this situation (Fieberg et al., 2018). Thus, we used calibration plots designed for use‐availability data (Boyce et al., 2002; C. J. Johnson et al., 2006) to further evaluate model performance. For each fold in a seasonal model set, we binned the testing data using quantiles of predicted probabilities and determined the predicted number of used points in each bin and the observed (Boyce et al., 2002; Fieberg et al., 2018; C. J. Johnson et al., 2006).We evaluated correlation between the expected and observed values using Spearman's rank correlation (Boyce et al., 2002).

We mapped model predictions across the state to quantify habitat selection across the landscape in each season. As with any resource selection function, the probability of a map pixel belonging to the “used” category is proportional to true probability of use by an unknown constant (Manly et al., 2002). This constant is model‐specific, which makes predictions not directly comparable across models. To make the output of our seasonal models directly comparable, we calculated relative selection strength (RSS; Avgar et al., 2017) by dividing predicted probabilities of use by the probability of use of a hypothetical pixel where all predictors are fixed at their mean seasonal value. The resulting metric expresses preference (if > 1) or avoidance (if < 1) of a pixel versus the average conditions observed across the landscape in each season. For our seasonal maps, we averaged values of RSS across folds. We also summarized results across seasons into a year‐round habitat/nonhabitat map and obtained by assigning any pixel with RSS > 1 in at least one season to habitat and any pixel with all seasons < 1 to nonhabitat (using the average value of RSS across folds.)

We assessed the importance of covariates in predicting habitat use by sage‐grouse based on the mean decrease in node impurity (MDI; Calle & Urrea, 2011). Unlike other variable importance metrics, MDI is robust to variable correlation, which was a concern in our case (Appendix). Finally, to visualize responses to important predictors, we calculated RSS across the range of values of each predictor with respect to its average value, while holding the value of all other predictors fixed at the mean (we used seasonal means for climate variables).

## 3 RESULTS

**FIGURE 1:**
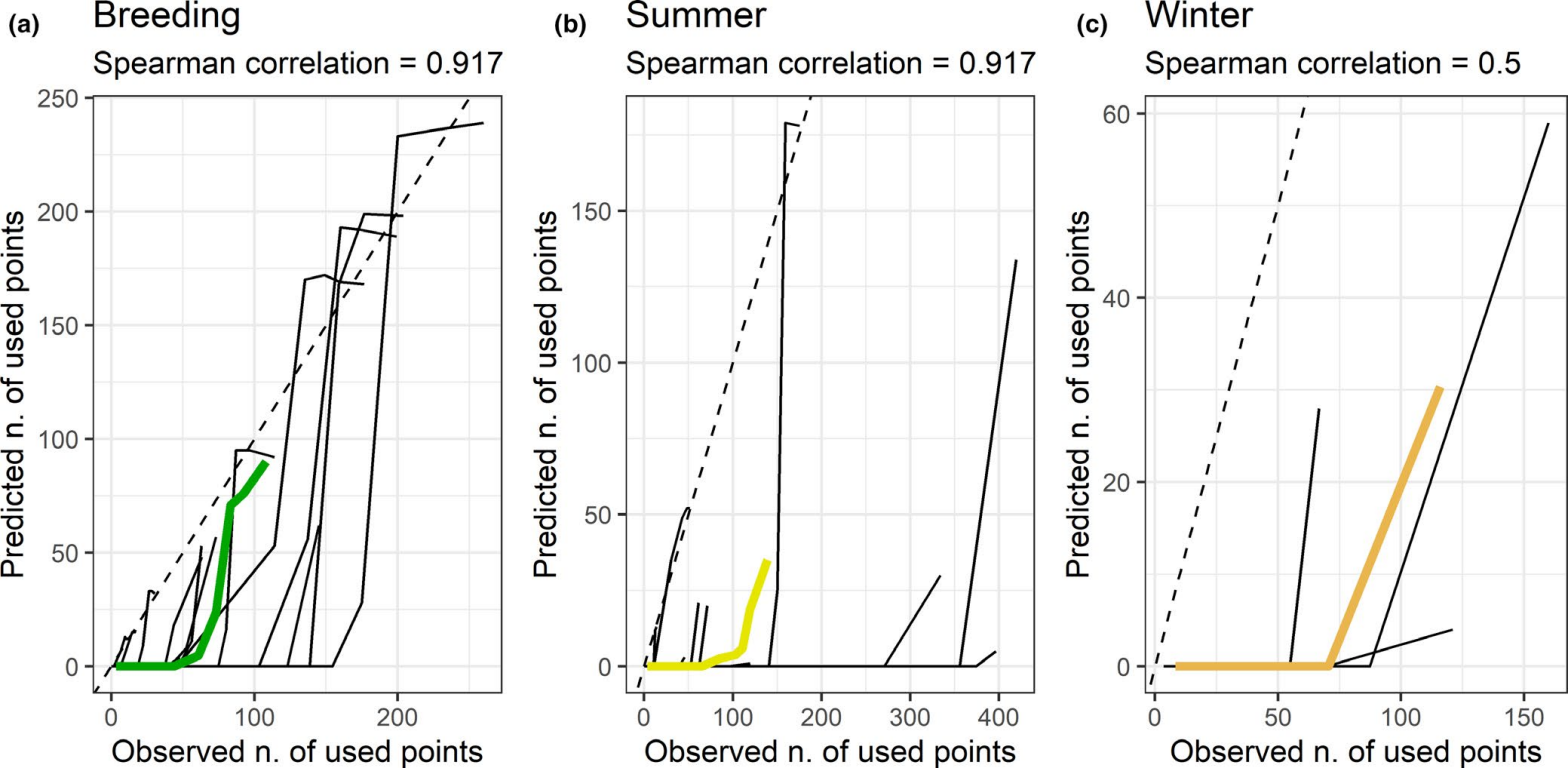
Calibration plots for random forest model of seasonal habitat selection for sage‐grouse. The solid black curves show the correlation between predicted and observed number of used points within each quantile of predicted probabilities for each of the K cross‐validation folds (following recommendations in Boyce et al., 2002). The colored curves depict seasonal means across folds. The dashed lines indicate the ideal 1:1 correlation, for reference. a) Breeding, b) Summer, c) Winter

The final dataset we used for our analysis included 6,885, 9,501, and 3,253 used locations for breeding, summer, and winter, respectively. For all models, out‐of‐bag classification errors were between 0.02 and 0.03 (Table 2). Classification errors based on spatial K‐fold cross‐validation were between 0.07 and 0.29 (Table 2). Spearman correlation coefficients for model calibration were 0.92 for breeding, 0.92 for summer, and 0.50 for winter (Figure 1).

**TABLE 2:** Out‐of‐bag and validation data confusion matrices for seasonal models of sage‐grouse habitat selection. We only calculated classification error rates for the used class because under a presence‐background study design available points are not necessarily unused; rather, their status is unknown

	Out‐of‐bag				Validation data			
		Used	Available	Class error		Used	Available	Class error
Breeding	*Used*	81,196	1,424	0.02	*Used*	3,168	218	0.07
Summer	*Used*	112,153	1859	0.02	*Used*	713	136	0.19
Winter	*Used*	31,727	803	0.03	*Used*	338	97	0.29

**FIGURE 2:**
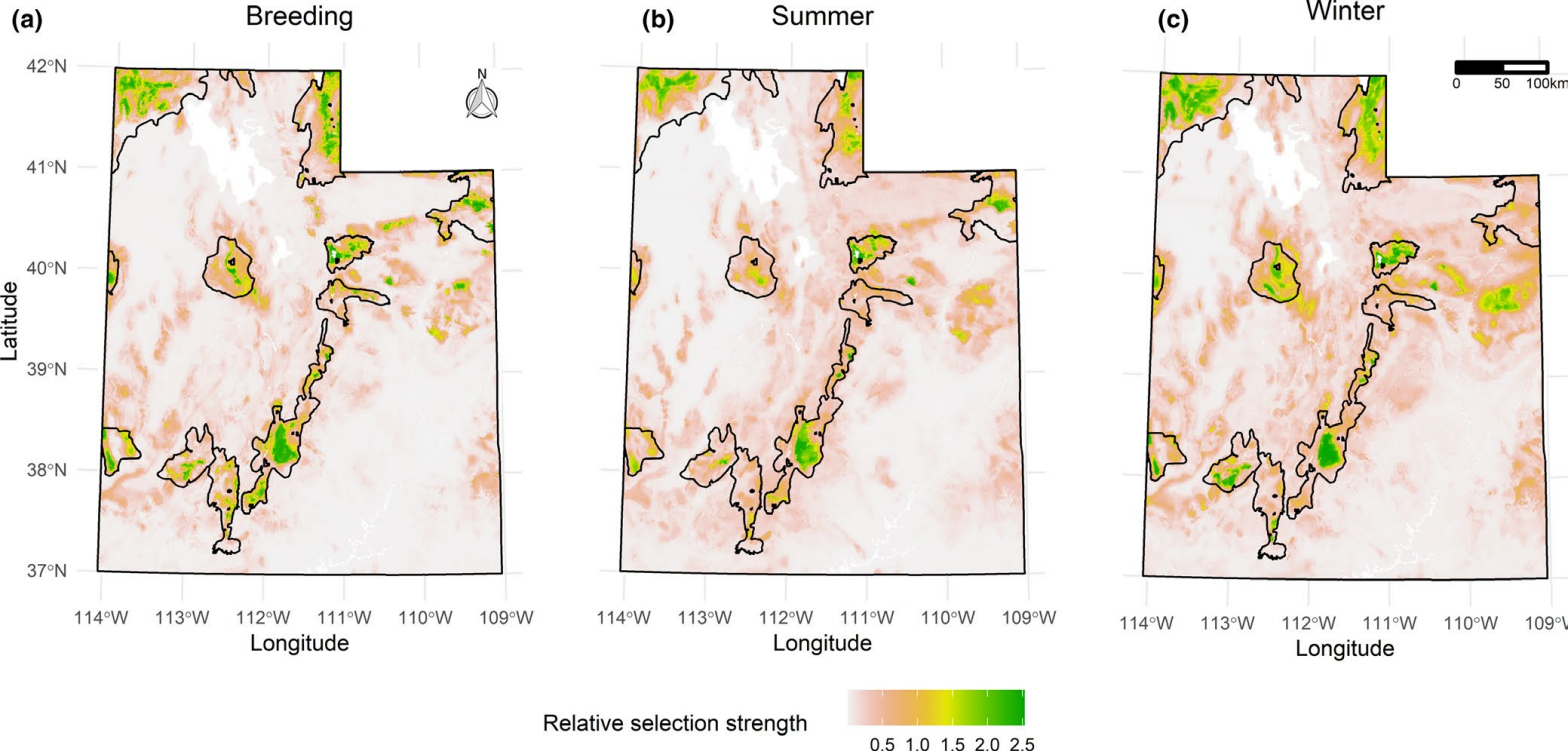
Seasonal habitat selection maps for greater sage‐grouse in the state of Utah. The color gradient shows relative selection strength, with values > 1 indicating selection over average conditions and values < 1 indicating avoidance. The black polygons depict existing Sage‐Grouse Management Areas. a) Breeding, b) Summer, c) Winter

**FIGURE 3:**
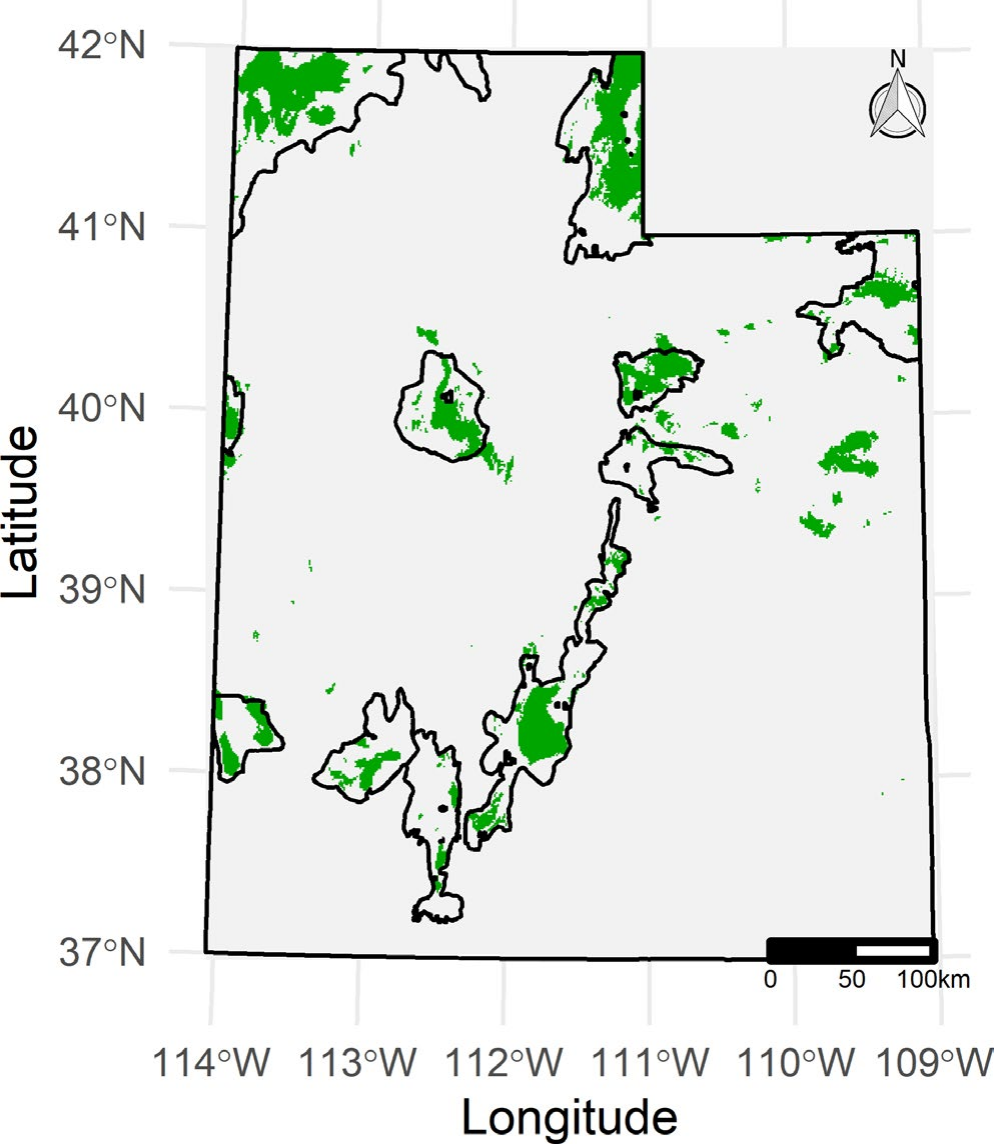
Map summarizing greater sage‐grouse habitat selection at the annual scale. Green indicates relative selection strength > 1 compared to average conditions when summing across seasons, white indicates < 1

**FIGURE 4:**
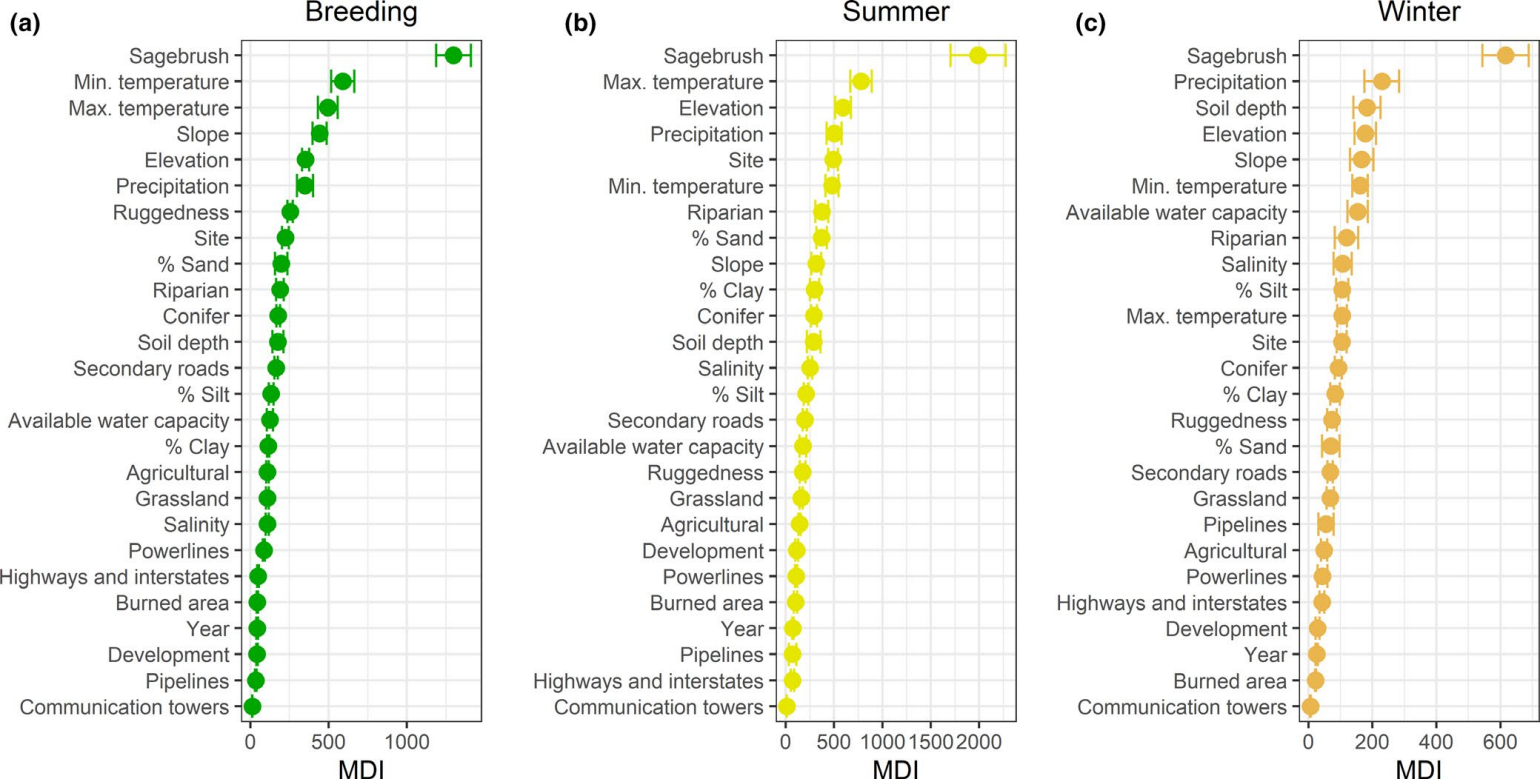
Variable importance plots based on mean decrease in impurity. Node impurity is measured by the Gini index. Variables are ranked following decreasing importance for sage‐grouse habitat selection in each season. The points depict mean values of MDI across the K cross‐validation folds and the bars indicate the standard deviation across folds. a) Breeding, b) Summer, c) Winter

Spatially explicit predictions of habitat selection for sage‐grouse aligned with known areas of occupied habitat throughout the state and generally followed Utah SGMA boundaries, with 83% of modeled annual habitat falling within SGMAs (Figures 2 and 3). Percent sagebrush cover in the surrounding 5 km radius was the most important predictor of sage‐grouse habitat selection in all seasons (Figure 4). Other predictors that were consistently highly ranked included slope, elevation, mean annual precipitation, and average minimum and maximum temperature; their order varied between seasons (Figure 3). Disturbance variables, especially communication towers and pipelines, were ranked as least important across seasons (Figure 4).

**FIGURE 5:**
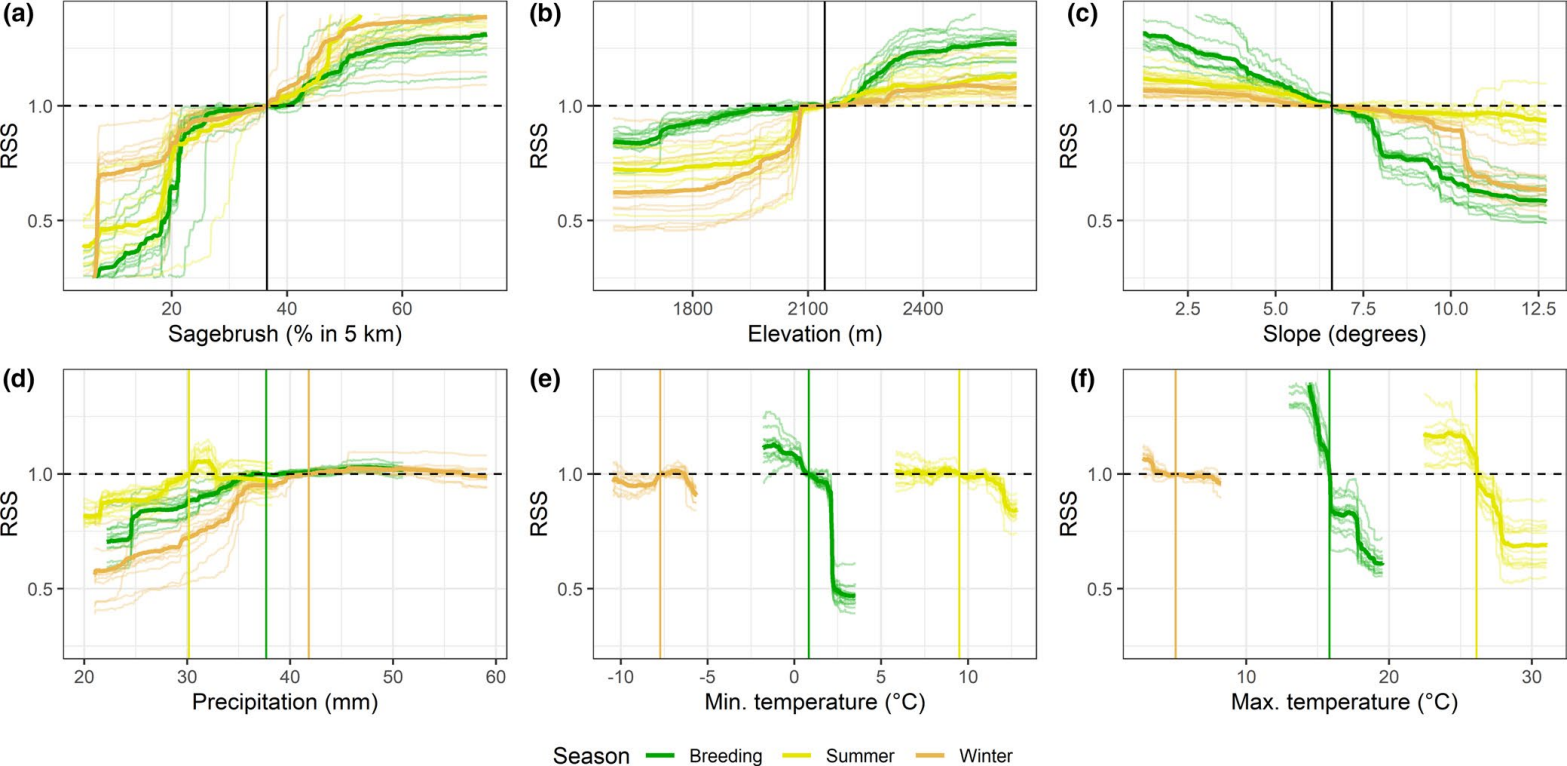
Model predictions for each of the top‐ranked six variables according to MDI across seasons. The dashed line divides selection (above) from avoidance (below). The vertical line marks the mean value of the predictor, against which relative selection strength is calculated (seasonal means in the case of climate variables, overall means for all others.) The bold line indicates predictions averaged across the K cross‐validation folds, while the shaded lines report individual estimates for each fold. Values in the top‐right quadrant of each plot indicate selection for values above the mean; top‐left, selection for values below the mean; bottom‐left, avoidance of values below the mean; bottom‐right, avoidance of values above the mean. a) Sagebrush; b) elevation; c) slope; d) mean annual precipitation (30‐year seasonal average); e) average minimum temperature (30‐year seasonal average); f) average maximum temperature (30‐year seasonal average)

Sage‐grouse selected habitat with higher‐than‐average percent sagebrush cover in all seasons and avoided lower‐than‐average sagebrush cover (Figure 5a). They selected for higher‐than‐average elevations (especially in the summer and during breeding) and strongly avoided lower‐than‐average ones (especially in winter and, to a lesser extent, summer; Figure 5b). They selected for gentler‐than‐average slopes (especially during breeding) and avoided steeper‐than‐average ones in breeding and winter (Figure 5c). They avoided areas with lower‐than‐average mean precipitation, especially during breeding and summer, when they also selected for higher‐than‐average precipitation, whereas during winter they avoided the highest precipitation values after reaching an optimum (Figure 5d). They favored areas with lower‐than‐average mean temperatures (both maximum and minimum; Figure 5e); they avoided areas with higher‐than‐average minimum temperatures, especially during breeding and summer, and selected for those with lower‐than‐average maximum temperatures in all seasons (Figure 5f).

## 4 DISCUSSION

We quantified and mapped seasonal second‐order habitat selection for sage‐grouse across the state of Utah, which constitutes the southern periphery of the species range (Stiver, 2006). Sage‐grouse in Utah selected for seasonal habitat where sagebrush covered a large proportion of the surrounding landscape, highlighting that the paramount role of sagebrush in determining sage‐grouse distribution holds constant across scales. However, we also found that sage‐grouse habitat selection in Utah differed from patterns previously reported at the range‐wide scale (Doherty et al., 2016; Knick et al., 2013). In particular, we found selection for higher elevation and higher precipitation compared to range‐wide estimates. We also found a weaker effect of anthropogenic development on sage‐grouse occupancy than what has been reported range‐wide (Knick et al., 2013). Our results indicate that not all habitat relationships that occur at the range‐wide scale apply at finer scales, and this highlights the importance of refining our understanding of sage‐grouse habitat selection within state jurisdictions (especially those at the periphery of the range) to effectively support management.

Model evaluation metrics indicated good performance of the random forest in correctly classifying used points from an independent subset of data spatially uncorrelated with the training data (Table 2; Figure 1). The spatial K‐fold cross‐validation classification error was especially low for the breeding season. The Spearman correlation coefficient from the calibration plots was >0.9 for both breeding and summer. Unsurprisingly, classification errors from spatial K‐fold cross‐validations were higher than out‐of‐bag errors: differences among sites across Utah are sometimes quite striking, and habitat models are known to inevitably exhibit a loss of performance when predicting over areas that were not represented in the training data (Ploton et al., 2020). Our spatial K‐fold cross‐validation approach reinforced our expectation that sage‐grouse responses to habitat variables would differ between study sites. Site was ranked as the fifth most important variable in our summer model, eighth in the breeding model, and twelfth in the winter model (Figure 4). When visualizing responses to individual variables (Figure 5), looking at the range of variation in model predictions across folds (each of which is built based on data from all sites except one) provides an indirect but useful indication of the breadth of variation in responses across sites.

One factor contributing to the poorer performance of the winter model compared to breeding and summer was the smaller dataset available for this season (both in terms of number of locations and sites surveyed). Moreover, we acknowledge that the nature of VHF data is necessarily limited by practical constraints, with more accessible locations being more likely to be recorded, which can introduce sampling bias and homogenize the habitat features that tracked individuals may have selected for. Harsh working conditions during the winter may have exacerbated this sampling bias by further constraining the accessibility of some areas for survey, possibly reducing the information content in an already smaller dataset. Another factor contributing to the relatively poor performance of the winter models may be that environmental differences among study sites at different latitudes or elevations become more extreme during winter, affecting the model's ability to extrapolate predictions over unsampled areas in the spatial K‐fold cross‐validation. Nonetheless, over 70% of used points were correctly classified using spatially independent testing data even in winter (and up to 93% for breeding).

Eighty‐three percent of habitat that our model identified as selected over average conditions fell largely within the Utah SGMA boundaries (Figures 2 and 3). Similarly, Dahlgren et al. (2016) reported that Utah's SGMAs included approximately 85% of the VHF radio‐telemetry seasonal locations and > 95% when weighted by lek counts. As we also expected, sage‐grouse habitat selection in SGMAs varied by season (Figure 2) and responses to individual landscape variables further confirmed and helped characterize seasonal differences in habitat selection (Figure 5). The selection for high elevation and gentle slopes and avoidance of areas with high minimum temperature we found during the breeding season is consistent with sage‐grouse biology in this part of their range. In Utah, leks are located at mid‐elevations where little or no slope occurs and more contiguous patches of sagebrush are typically found, and nesting activities are concentrated near leks (Dahlgren et al., 2016, 2019). Selection for higher elevations with low maximum temperature and avoidance of low precipitation during the summer is also consistent with sage‐grouse biology. Higher precipitation drives the growth of annual and perennial forbs, which in turn support high densities of arthropods, both of which provide food for sage‐grouse chicks during brood‐rearing periods (Klebenow & Gray, 1968). The patterns of winter habitat selection we observed (weaker selection for higher elevation and avoidance of steeper slopes, low precipitation, and low minimum temperatures) reflect the need for sage‐grouse to balance forage intake and overhead cover with reduced exposure to weather conditions. During the winter, sage‐grouse depend on sagebrush plants exposed above the snow for food and cover (Connelly et al., 2000; Dahlgren et al., 2019); therefore, the milder climatic conditions with less snow accumulation make lower‐elevation areas more desirable in this season (Connelly, et al., 2011; Crawford et al., 2004; K. T. Smith et al., 2014). In addition, soil depth was ranked as an important variable for predicting sage‐grouse habitat selection in winter, whereas it did not appear among the top variables for either breeding or summer. Specifically, sage‐grouse avoided areas with lower‐than‐average soil depth (Appendix). Deep soils are suitable for growth of several subspecies of big sagebrush (Rosentreter, 2004), some of which are highly palatable to sage‐grouse and a major food source during the winter, when the chemical content of the leaves becomes sufficiently low and their availability also becomes limiting due to snow cover (Roberson, 1986; Welch et al., 1988; Welch et al., 1991; Wing & Messmer, 2016).

Consistent with the fact that habitat selection manifests differently at different hierarchical levels, the features we found sage‐grouse select for at the second order partially differed from what drives their broad‐scale distribution (Doherty et al., 2016; Knick et al., 2013). When comparing our findings with the range‐wide results presented by Knick et al. (2013), sage‐grouse in Utah appear to select for higher elevations, lower temperatures, and higher precipitations than they do at the range‐wide scale. However, their approach to developing a species distribution model for sage‐grouse was based on the concept of limiting factors (or “ecological minimums,” Knick et al., 2013), and therefore, they were interested in similarities among used locations rather than differences between used and available; consequently, they used a different analytical approach than the one we employed, which may confound direct comparisons between our results. Using a similar methodology to ours, Doherty et al. (2016) found that, across management zones throughout the sage‐grouse species range, elevation was not a strong predictor of sage‐grouse habitat selection. The differences we reported with studies at broader scales likely reflect the specific and unique geographical and topographical context of our study area at the southern periphery of the sage‐grouse range (Dahlgren et al., 2016). The geography of Utah is characterized by mountainous terrain, separated by broad valleys in the Great Basin and by deeply incised canyons in the Colorado Plateau (West, 1983). The vast, relatively low‐elevation patches of sagebrush steppe found elsewhere across the sage‐grouse range are less common in Utah than in other parts of the species range. With the exception of populations in Box Elder, Rich, and Uinta county, which occur in contiguous sagebrush steppe extending into neighboring states, sage‐grouse in Utah inhabit smaller remnant fragments of sagebrush at high elevations (Dahlgren et al., 2016). Thus, elevation was the most important factor in distinguishing sage‐grouse habitat characteristics in Utah (Dahlgren et al., 2019). Differences in elevation are also correlated with differences in climatic regimes; hence, the patterns we found in terms of temperature and precipitation when comparing sage‐grouse habitat selection in Utah with the rest of the range (Miller & Eddleman, 2000).

Anthropogenic disturbances were found to be major drivers affecting sage‐grouse distribution at the range‐wide scale (Doherty et al., 2016; Knick et al., 2013). However, we found some of the anthropogenic variables previously reported as important to be relatively low‐ranked predictors of sage‐grouse habitat selection in Utah. Our results may reflect differences in habitat selection across hierarchical scales (second‐ versus first‐order), as well as reflect the spectrum of conditions that occur in Utah as compared to the species range (Stiver, 2006). Levels of agricultural disturbance within occupied sage‐grouse habitats in Utah are relatively low compared to other areas range‐wide (Connelly et al., 2004; Gibson et al., 2014). Similarly, the energy development footprint on sage‐grouse habitat within Utah is relegated to the northeastern part of the state (Doherty, 2008). Given these conditions, avoidance of disturbance may not emerge as a substantial factor driving sage‐grouse habitat selection in Utah, but may occur at even finer scales such as those within a given sage‐grouse population. Based on these results, we reiterate that conservation planners should be wary of extrapolating habitat relationships beyond the scale, spatial domain, and ecological context in which they were first delineated (J. T. Smith et al., 2020). Finally, we underline that variable importance results should not be interpreted in absolute terms, but only relative to each other. Relatively low‐ranked variables may still play a role in shaping habitat selection patterns, but the contribution they bring to the model in terms of predictive power is smaller compared to higher‐ranked variables.

Somewhat surprisingly, conifer cover did not appear among the top‐ranked variables in any of our models, despite conifer encroachment negatively impacts sage‐grouse survival (Coates et al., 2017), sage‐grouse lek activity decreases with increasing conifer stand cover (Baruch‐Mordo et al., 2013), and sage‐grouse select for areas where conifer removal treatments have been implemented (Cook et al., 2017; Sandford et al., 2017). The relationship we found with conifer indicated slight selection for areas with no conifer cover and moderate avoidance of areas with high conifer cover (Appendix), which is consistent with previous evidence. However, the relatively low ranking we observed for the importance of conifer cover in our models might depend on the fact that the variable does not distinguish between conifer encroachment and conifer forests per se.

Our models agreed with the range‐wide models that the proportion of surrounding landscape occupied by sagebrush cover is the single most important predictor of sage‐grouse occurrence across scales (Doherty et al., 2016; Knick et al., 2013). Although this result was expected, it corroborates the growing body of scientific evidence that conserving large, connected, and contiguous areas of sagebrush is necessary to sustain sage‐grouse populations in the future (Aldridge et al., 2008; Crawford et al., 2004; Knick et al., 2013; J. T. Smith et al., 2020). Despite being fragmented at the state‐wide level, the sagebrush ecosystems found in Utah can sustain viable sage‐grouse populations, especially if connectivity between populations is maintained by implementing habitat conservation and restoration actions such as management of pinyon‐juniper (*Pinus* spp. and *Juniperus* spp.) encroachment and cheatgrass (*Bromus tectorum*) invasion (Connelly et al., 2000; Cook et al., 2017; Crawford et al., 2004; Dahlgren et al., 2016; Sandford et al., 2017). However, further research will be needed to identify thresholds for sagebrush patch sizes that render them insufficient for providing habitat or enhancing connectivity.

Our results highlight the importance of conducting state‐specific studies to support decisions that are made by management agencies at the state level, adopting a multi‐scale approach to conservation planning (Doherty et al., 2010). Because scale is important biologically, it is important for management: to effectively support conservation decisions, evidence must be scale‐appropriate (Ferraz et al., 2012). Several studies have emphasized the importance of integrating analyses from different spatial scales to effectively inform management of species of conservation concern (Bergman et al., 2012; McGarigal et al., 2016; Mills et al., 2010; Wiens & Bachelet, 2010); our findings provide an example of how considering the hierarchical nature of ecological processes can lead to thoughtful consideration of which scale is appropriate for the objective at hand and result in more refined information and tailored solutions to conservation problems.

## COMPETING INTERESTS STATEMENT

We declare we have no competing interests.

## AUTHOR CONTRIBUTION

**Simona Picardi:** Formal analysis (lead); Methodology (lead); Software (equal); Visualization (lead); Writing‐original draft (lead). **Terry A Messmer:** Conceptualization (equal); Project administration (lead); Resources (equal); Supervision (lead); Writing‐review & editing (lead). **Ben Crabb:** Conceptualization (equal); Data curation (lead); Formal analysis (supporting); Methodology (supporting); Software (equal); Writing‐review & editing (lead). **Michel Kohl:** Data curation (supporting); Writing‐review & editing (lead). **David K. Dahlgren:** Resources (equal); Writing‐review & editing (equal). **Shandra N. Frey:** Resources (equal); Writing‐review & editing (equal). **Randy T Larsen:** Resources (equal); Writing‐review & editing (equal). **RICK BAXTER:** Resources (equal); Writing‐review & editing (equal).

## Supporting information

Appendix FigureA1–A4

## Data Availability

The sage‐grouse is a species of national and state conservation concern. The Utah lek location data used to conduct our research are protected by Utah state law. We requested these data from Utah Division of Wildlife Resources (UDWR) through a Government Records Access and Management Act (GRAMA) request for the specific purposed of the research. Others requesting access to these data will have to GRAMA the UDWR. Similarly, the sage‐grouse radio‐telemetry data used to complete this research are also protected by confidentiality agreements with landowners and managers who allowed the researchers access to landscapes for the purposes of the research.
