# Supplementary material for: Predicting greater sage‐grouse habitat selection at the southern periphery of their range
Source: Ecol Evol. 2020 Oct 28;10(23):13451–63. doi: 10.1002/ece3.6950 (PMC7713982; doi:10.1002/ece3.6950)
Supplement: Supplementary file 1 — Appendix FigureA1–A4 [file ECE3-10-13451-s001.docx]

**Predicting Habitat Suitability for Greater Sage-Grouse at the Southern Periphery of their Range**

**Appendix**

**
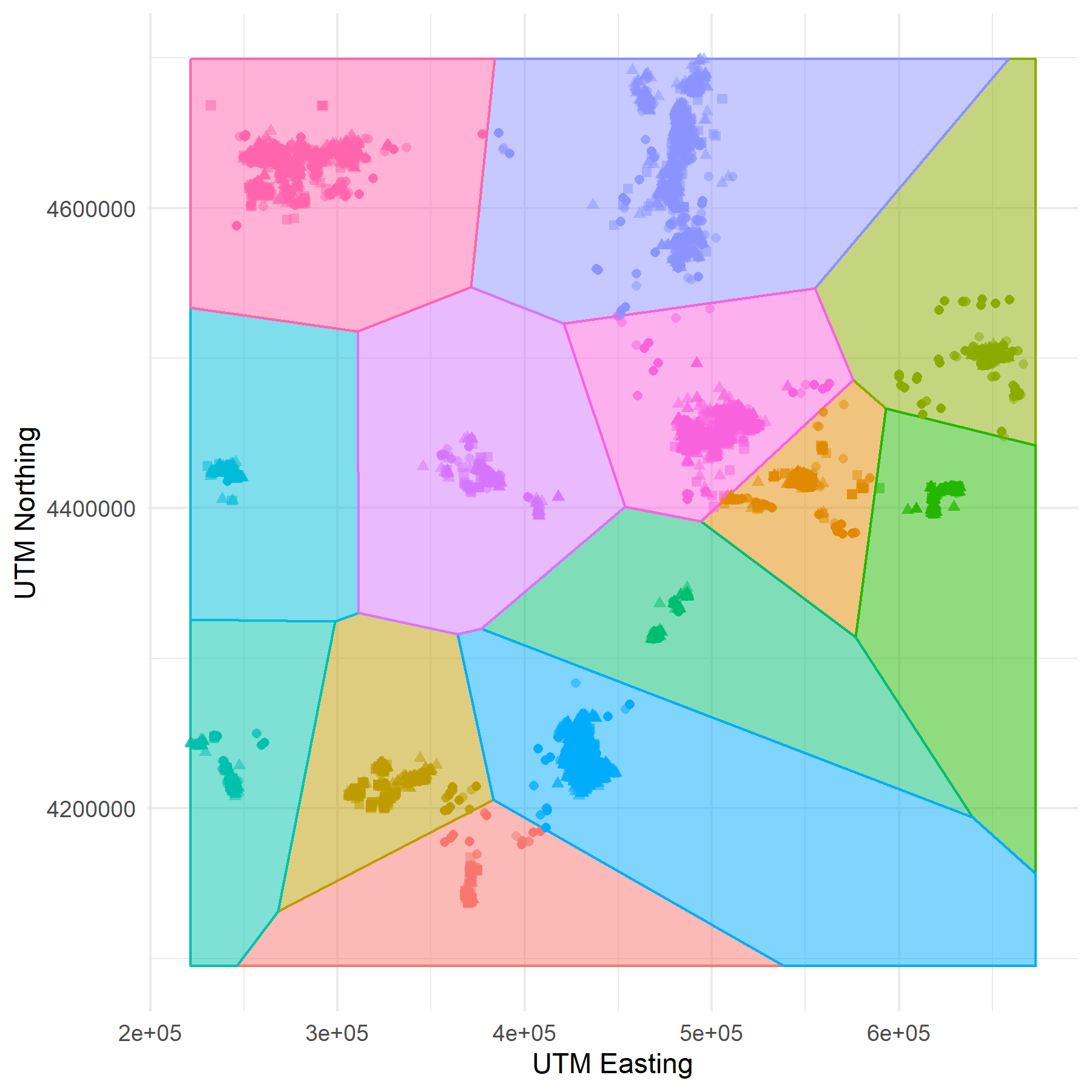
**

Figure 1. Voronoi polygons built around the centroids of sage-grouse populations (i.e., populations inhabiting study areas showing marked difference in terms of environmental characteristics, and/or separated by land where sage-grouse do not occur) used to assign a site to each location. Used sage-grouse locations are overlaid with different symbols for each season (circles for breeding, triangles for summer, and squares for winter).


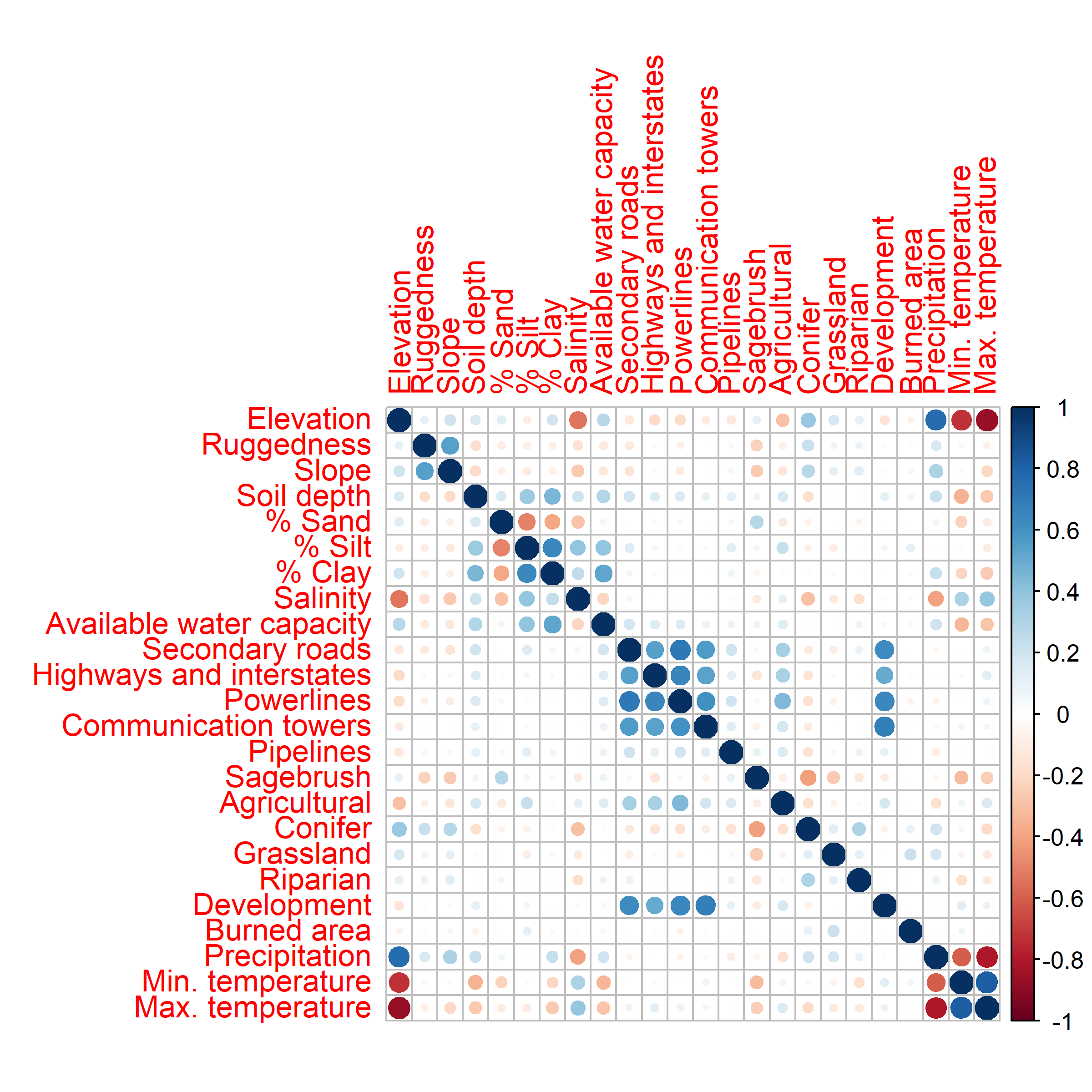


Figure 2. Correlation plot for variable included in sage-grouse habitat selection models. The size of the dots indicates the strength of correlation and the color indicates the direction of the correlation (blue for positive, red for negative).


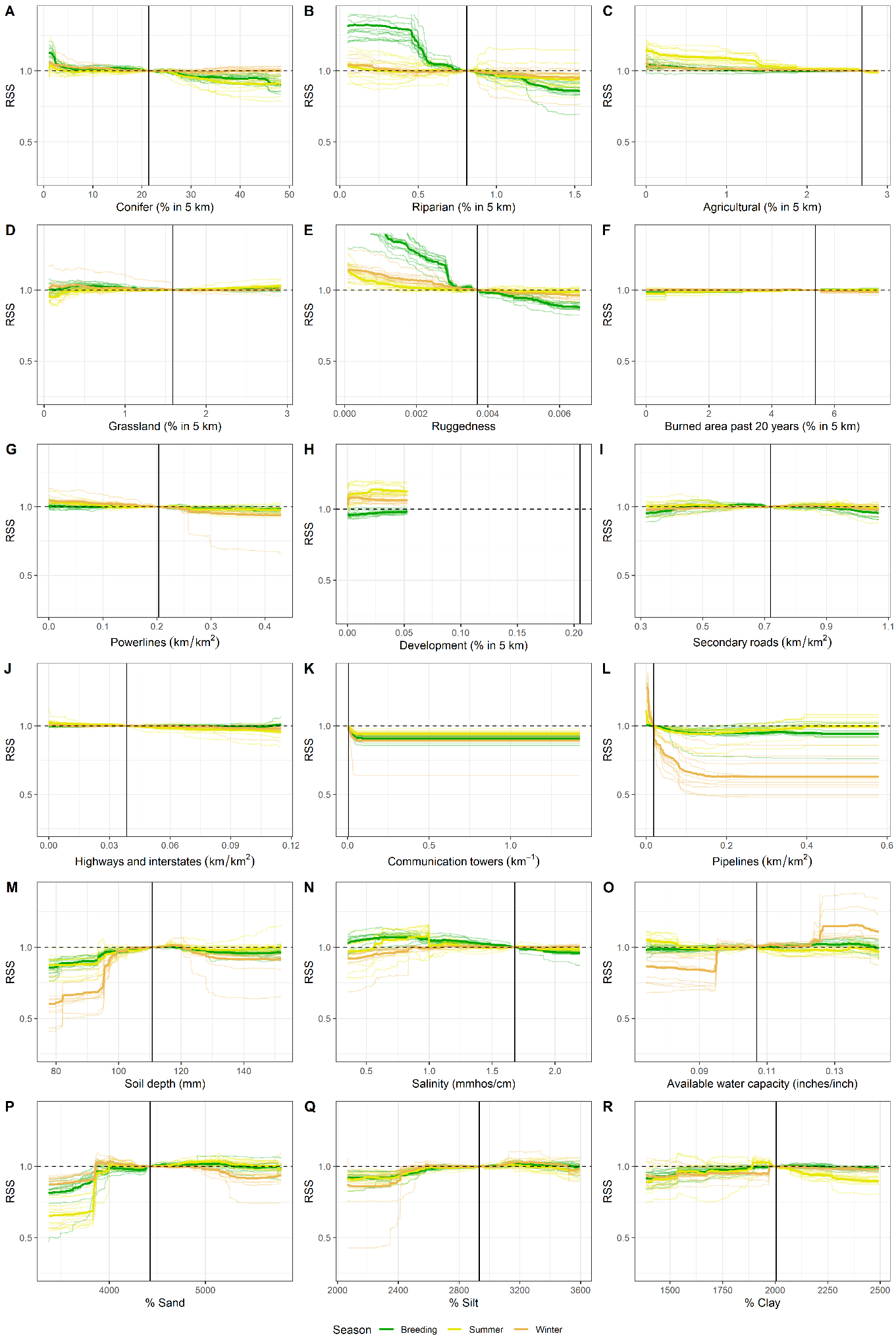


Figure 3. Model predictions for each of the remaining variables included in our models but not represented in Figure 5. A) Conifer cover, B) Riparian habitat, C) Agricultural land, D) Grassland, E) Ruggedness, F) Burned area within the past 20 years, G) Powerlines, H) Development, I) Secondary roads, J) Highways and interstates, K) Communication towers, L) Pipelines, M) Soil depth, N) Salinity, O) Available water capacity, P) Percent sand, Q) Percent silt, R) Percent clay. These variables were consistently ranked below the top six in our results (except soil depth (M) in winter). The dashed line divides selection (above) from avoidance (below). The vertical line marks the mean value of the predictor, against which relative selection strength is calculated. The bold line indicates predictions averaged across the K cross-validation folds, while the shaded lines report individual estimates for each fold. Values in the top-right quadrant of each plot indicate selection for values above the mean; top-left, selection for values below the mean; bottom-left, avoidance of values below the mean; bottom-right, avoidance of values above the mean.

**
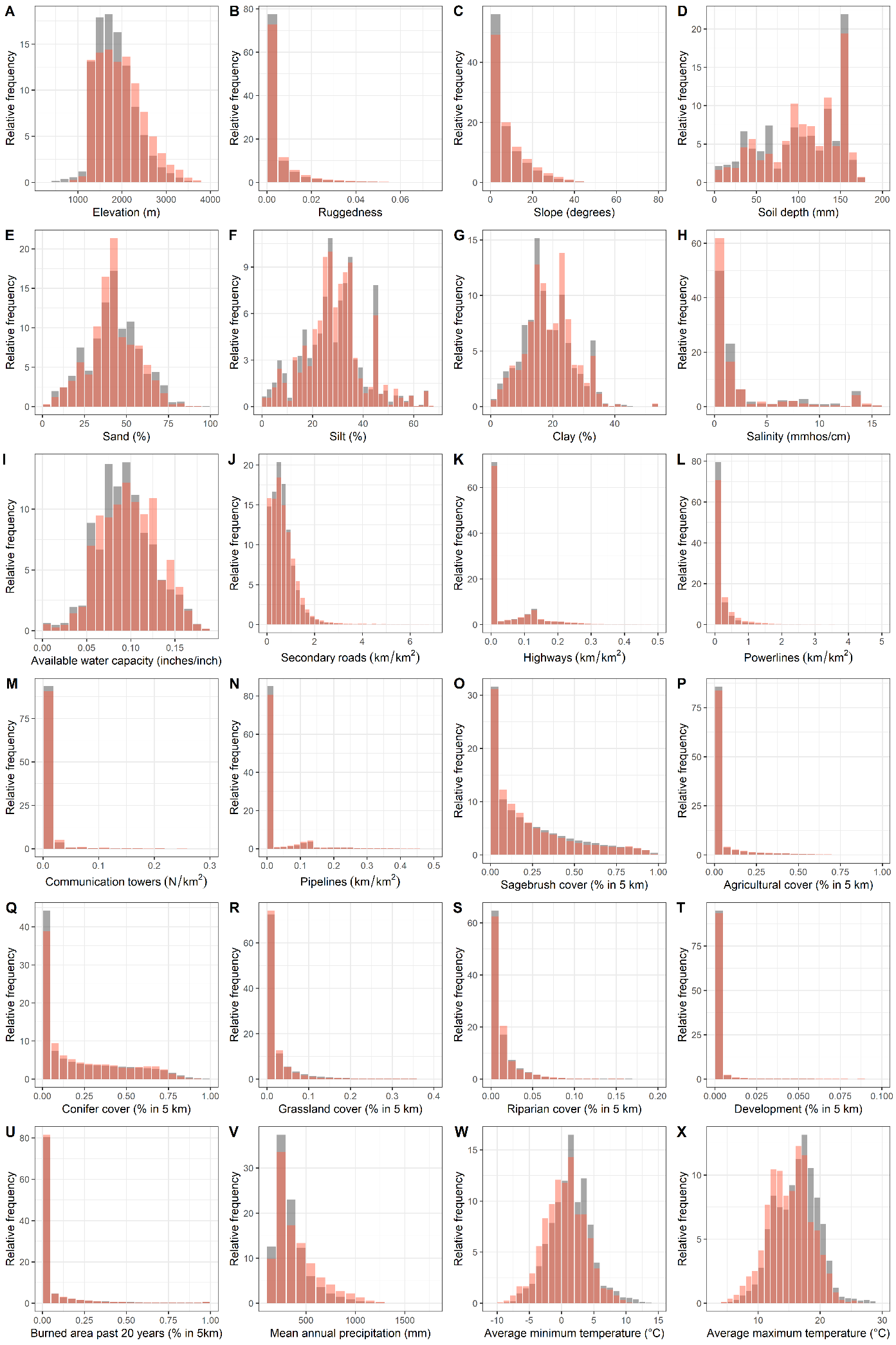
**

Figure 4. Histograms of variables included in the model; gray bars indicate distribution across the entire state, shaded red bars indicate the availability domain captured by the sample of available points included in the model. Perfectly overlapping histograms would indicate that the sample of available points included in the model perfectly represents the range of available conditions across the study area. A) Elevation; B) Ruggedness; C) Slope; D) Soil depth; E) Percent sand; F) Percent silt; G) Percent clay; H) Salinity; I) Available water capacity; J) Secondary roads; K) Highways and interstates; L) Power lines; M) Communication towers; N) Pipelines; O) Sagebrush cover; P) Agricultural cover; Q) Conifer cover; R) Grassland cover; S) Riparian cover; T) Development; U) Burned area; V) Mean annual precipitation; W) Average minimum temperature; X) Average maximum temperature.
